# Response of a simian immunodeficiency virus (SIVmac251) to raltegravir: a basis for a new treatment for simian AIDS and an animal model for studying lentiviral persistence during antiretroviral therapy

**DOI:** 10.1186/1742-4690-7-21

**Published:** 2010-03-16

**Authors:** Mark G Lewis, Sandro Norelli, Matt Collins, Maria Letizia Barreca, Nunzio Iraci, Barbara Chirullo, Jake Yalley-Ogunro, Jack Greenhouse, Fausto Titti, Enrico Garaci, Andrea Savarino

**Affiliations:** 1 BIOQUAL, Inc 9600 Medical Center Drive, Rockville, MD 20850, USA; 2 Department of Infectious, Parasitic and Immune-mediated Diseases, Istituto Superiore di Sanità, Viale Regina Elena, 299, 00161, Rome, Italy; 3 Dipartimento di Chimica e Tecnologia del Farmaco, Facoltà di Farmacia, Università di Perugia, Via del Liceo 1, 06123, Perugia, Italy; 4 CNAIDS, Istituto Superiore di Sanità, Viale Regina Elena, 299, 00161, Rome, Italy; 5 Department of Experimental Medicine, University of Rome Tor Vergata, Rome, Italy

## Abstract

**Background:**

In this study we successfully created a new approach to ART in SIVmac251 infected nonhuman primates. This drug regimen is entirely based on drugs affecting the pre-integration stages of replication and consists of only two nucleotidic/nucleosidic reverse transcriptase inhibitors (Nt/NRTIs) and raltegravir, a promising new drug belonging to the integrase strand transfer inhibitor (INSTI) class.

**Results:**

In acutely infected human lymphoid CD4^+ ^T-cell lines MT-4 and CEMx174, SIVmac251 replication was efficiently inhibited by raltegravir, which showed an EC_90 _in the low nanomolar range. This result was confirmed in primary macaque PBMCs and enriched CD4^+ ^T cell fractions. *In vivo *monotherapy with raltegravir for only ten days resulted in reproducible decreases in viral load in two different groups of animals. When emtricitabine (FTC) and tenofovir (PMPA) were added to treatment, undetectable viral load was reached in two weeks, and a parallel increase in CD4 counts was observed. In contrast, the levels of proviral DNA did not change significantly during the treatment period, thus showing persistence of this lentiviral reservoir during therapy.

**Conclusions:**

In line with the high conservation of the three main amino acids Y143, Q148 and N155 (responsible for raltegravir binding) and molecular docking simulations showing similar binding modes of raltegravir at the SIVmac251 and HIV-1 IN active sites, raltegravir is capable of inhibiting SIVmac251 replication both in tissue culture and *in vivo*. This finding may help to develop effective ART regimens for the simian AIDS model entirely based on drugs adopted for treatment in humans. This ART-treated AIDS nonhuman primate model could be employed to find possible strategies for virus eradication from the body.

## Background

Integration of proviral DNA into the host's genome is a fundamental step in lentiviral infections, initiating the latency period, and allowing the virus to exploit the cellular transcriptional and translational machinery [1,2]. The recent approval of the integrase strand transfer inhibitor (INSTI) raltegravir for first-line HIV-1 therapy thus provides a further option for treatment of drug-naïve HIV-1 infected patients [3]. INSTIs selectively inhibit the strand transfer reaction, catalyzed by HIV-1 integrase (IN) after 3' processing, which generates a reactive 3'-hydroxylgroup in proviral DNA. Raltegravir represents a major success in the history of antiretroviral therapy (ART) and is the result of a drug development process which encountered exceptional difficulties [1,4,5].

Despite this and other major successes in antiretroviral drug discovery and the availability of several drug options for obtaining sustained suppression of viral load in HIV-1 infected individuals, ART cannot eradicate the virus from the body [6], at least in a reasonable time [7]. The grounds for HIV-1 persistence during therapy lie in the presence of long-lived viral reservoirs (mainly the memory T CD4^+ ^cell subset), which harbour silent copies of proviral DNA that cannot be targeted by drugs or the immune system [6,8,9]. Alternative/complementary strategies are therefore being actively researched, in order to facilitate the purging of HIV-1 from reservoirs. To this end, the so-called "shock and kill" strategies have been proposed [8,10]. These strategies should induce, through drugs, HIV-1 activation from quiescence (*i.e*. the "shock" phase), in the presence of ART (to block viral spread), followed by the elimination of infected cells (*i.e*. the "kill" phase), through either natural means (*e.g*. immune response, viral cytopathogenicity) or artificial means (*e.g*. drugs).

One major obstacle which has been encountered by the studies on such "HIV-1 purging" strategies is the availability of reliable animal models. Such models should mimic the long-term effects of ART in humans. Interesting low-cost models include the new SCID mice technology [11] and feline immunodeficiency virus (FIV)-infected cats [12,13]; however, the macaque AIDS model has encountered the largest consensus in the AIDS researchers' community. This model is based on lentiviruses derived from African sooty mangabeys introduced into the non-natural host, Asian macaque species (*Macaca sp*.), which results in the development of illness similar to that described in AIDS patients [14]. Recently, also chimpanzees were found to develop disease when naturally infected with SIVcpz, the ancestor of HIV-1 group M [15]. However, the close phylogenetic relationships with humans restrict the use of these apes in the laboratory.

The simian AIDS model presents its own profile of response to HIV-1 drugs, rendering it difficult to treat with the ART protocols adopted for treatment of HIV-1/AIDS. For example, SIVmac251, one of the most commonly adopted viral strains for laboratory infection of macaques, is fully sensitive to nucleotidic and nucleosidic reverse transcriptase inhibitors (NtRTIs/NRTIs), retains limited sensitivity to some, but not all of the protease inhibitors (PIs) designed for HIV-1, and shows approximately 200-fold less sensitive to non-nucleosidic reverse transcriptase inhibitors [16]. Treatment with NtRTI tenofovir (also referred to as PMPA) and NRTI emtricitabine (FTC) represents a valuable option for studying the gene expression profiles activated during suppression of viral load and immune restoration [17]. However, this type of treatment can hardly be used to model long-term lentiviral persistence during ARTs designed for humans, which comprise three or more active drugs and at least two drug targets. The poor response of the laboratory simian lentiviruses to NNRTIs prompted some to replace the reverse transcriptase (RT) gene of the simian lentivirus with a gene encoding HIV-1 RT [18]. This substitution is extremely useful for studying the occurrence of drug resistance mutations *in vivo *[19], and for preclinical testing of novel NRTIs. However, an impact of the RT substitution on the natural history of the disease cannot be excluded so far. Indeed, apart from altering immunogenicity, replacement of a simian lentivirus's RT with its counterpart from HIV-1 might alter susceptibility of some cell populations to the virus. For example, RT-bound, elongating proviral DNA is the substrate of APOBEC3G, a species-specific cellular restriction factor to infection by primate lentiviruses [14]. Upon clarification of these issues, this simian/human immunodeficiency virus (SHIV) chimera could become an extremely useful tool to model ART consisting of two Nt/NRTIs and an NNRTI [20], a commonly adopted regimen for first-line treatment of HIV-1/AIDS.

New strategies for treatment of the macaque AIDS model may exploit the novel INSTI drug class. Hazuda *et al*. [21] evaluated, in SHIV 89.6P-infected rhesus nonhuman primates, the effects of naphthyridine carboxamide, L-870,812, an INSTI belonging to a chemical class distinct from that of raltegravir. This study provided the first proof of concept for an antiretroviral effect of IN inhibition *in vivo*. Moreover, L-870,812 monotherapy of macaques allowed the isolation of drug-resistant viruses presenting the N155H mutation, which later proved an important drug resistance mutation in HIV-1-infected individuals failing raltegravir-based regimes [22]. On this basis, an ART-treated nonhuman primate model was recently developed by Dinoso *et al*. using L-870,812 in combination with PMPAand two PIs, *i.e*. saquinavir and atazanavir in macaques infected simultaneously with SIV/17E-Fr and SIV/Delta B670 [23]. Sustained suppression of viral load was obtained until the end of follow-up. However, one limitation of this model is that this type of drug regimen is not adopted in humans. Moreover, the authors used two PIs at a relative dosage much higher than that adopted for humans.

The susceptibility of non-human primate lentiviruses to naphthyridine carboxamides is probably due to the high level of conservation of IN CCDs [12]. A three-dimensional (3D) structure [PDB: 1C6V] is available for the catalytic core domain (CCD) and C-terminal domain of the IN of SIVmac251 [24]. SIVmac251 IN catalyses reactions similar to those of HIV-1 IN, and the crystal structure shows that the IN of SIVmac251 shares the highly conserved three-dimensional (3D) architecture of retroviral INs [see Additional file 1] [24]. Accordingly, SIVmac251 has been reported to be susceptible also to the investigational HIV-1 INSTI, CHI/1043, belonging to the 1H-benzylindole drug class [25]. Despite this bulk of evidence, response of SIVmac251 to raltegravir has not yet been studied in detail. An extension of the data from other INSTI classes to raltegravir may not be obvious, because different classes of INSTIs may have different binding modes, as shown by the partially overlapping yet different drug resistance mutation profiles and molecular docking calculations [26,27].

The assessment of the response of a simian lentivirus laboratory strain to raltegravir may have important repercussions on the development of antiretroviral therapies for the simian AIDS model using a drug combination adopted in humans. Moreover, the response of a non-human lentivirus to this drug may furnish important insights into the requirements for susceptibility to this new and important drug class.

## Results

### Raltegravir inhibits SIVmac251 replication in tissue culture

**Figure 1:**
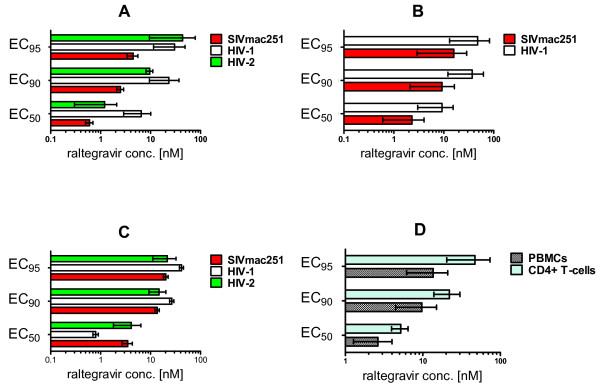
**SIVmac251 susceptibility to raltegravir in tissue culture**. The effective concentrations at 50%, 90% and 95% (respectively, EC_50_, EC_90_, and EC_95_) are presented (means ± SEM from at least two independent experiments) for inhibition of: lentiviral cythopathogenicity in MT-4 cells (Panel A), viral core antigen release in supernatants of acutely infected MT-4 cells (Panel B), syncytium formation in acutely infected CEMx174 cells (Panel C), viral core antigen release in supernatants of acutely SIVmac251-infected rhesus peripheral blood mononuclear cells (PBMCs) and enriched CD4^+ ^T-cell fractions (Panel D). In panel A, the inhibitory concentrations were determined by the methyl tetrazolium (MTT) method when the majority of control infected cells (in the absence of drug treatment) were dead at light microscopy examination. In panel B, values were derived by quantifying, using antigen-capture ELISA assays, SIVmac251 p27 and HIV-1 p24 in supernatants from five-day old cultures. In panel C, values were calculated on the basis of the numbers of syncytia per well at five days post-infection, Syncytia were counted in triplicate on three different occasions by light microscopy. In panel D, values are representative for supernatants of primary cells from three different donors at Day 5 post-infection.

To test susceptibility of SIVmac251 to raltegravir, MT-4 cells were infected with SIVmac251, washed and incubated with decreasing raltegravir concentrations. Response to raltegravir was assessed by the widely validated MTT assay, when the majority of cells in the untreated controls were dead, *i.e*., approx. fifteen days post-infection. Results showed that raltegravir inhibited SIVmac251 replication in the low nanomolar range (Fig. 1A). The EC_50 _was approximately one order of magnitude lower than that obtained using HIV-1 IIIB, which was calculated on the basis of data collected at five days post-infection due to the more rapid kinetics of viral cytopathogenicity (Fig. 1A). Data from HIV-2(strain: CDC 77618 [28])-infected MT-4 cell cultures showed intermediate characteristics between those obtained from SIVmac251- and HIV-1-infected cultures. Apart from being phylogenetically closer to SIVmac251 than to HIV-1, the HIV-2 strain that we used killed the majority of the infected cells in eight days following infection, thus showing viral cytopathogenicity kinetics slower than HIV-1 and more rapid than SIVmac251.

To assess whether the difference in the EC_50 _values for SIVmac251 and HIV-1 IIIB cytopathic effects were attributable to the different kinetics of viral cytopathogenicity, we measured, by antigen-capture ELISA assays, the viral core antigen in supernatants collected at five days post-infection from both the SIVmac251 and HIV-1 infected cell cultures. In this case, the ranges of the EC_50 _values for SIVmac251 and HIV-1 obtained in the different experimental set-ups were overlapping (Fig. 1B). We concluded that raltegravir inhibits SIVmac251 replication in human T-cell lines with similar potency as shown against HIV-1.

As different types of kits had to be used to compare inhibition of SIVmac251 p27 and HIV-1 p24 production, we decided to confirm the results using another method allowing simultaneous and homogeneous measurements of antiviral efficacy against SIVmac251, HIV-1, and HIV-2. We used syncytia counts in CEMx174 cells as a measure of lentiviral replication. SIVmac251 replication induces syncytia at an earlier time point as compared to the cytopathic effect induced in MT-4 cells, in which lentiviral replication mostly induces apoptotic and necrotic cell death [29]. The effectiveness of syncytia counts as a parameter for detection of the antiretroviral effects was confirmed by correlation analyses of syncytium formation and viral core antigen production in the presence of antiretroviral drugs (an example using raltegravir is given in the additional material [see Additional file 2]). CEMx174 cells were infected with SIVmac251, HIV-1, and HIV-2 viral stocks at the same multiplicity of infection (MOI), and syncytia were counted by optical microscopy at 4-5 days post-infection. Results confirmed that raltegravir exerted potent and reproducible anti-SIVmac251 activity (Fig 1C).

To assess the anti-SIVmac251 effects of raltegravir under conditions more closely resembling those occurring *in vivo*, 3 day-old PHA-stimulated peripheral blood mononuclear cells (PBMCs) from uninfected rhesus macaques (*Macaca mulatta*) were infected with SIVmac251, and viral replication was quantified in supernatants by ELISA at five days post-infection, in order to allow comparison with the results reported in the previous paragraph. Also in this case, raltegravir displayed an EC_50 _in the low nanomolar range (Fig 1D).

To assess the effect of raltegravir in the rhesus CD4^+ ^T cell population, *i.e*., the main target of SIVmac251 *in vivo*, we separated the CD4^+ ^T cells from fresh unstimulated PBMCs using magnetic beads. Flow cytometric analysis of the enriched CD4^+ ^T cell fraction showed that 94 to 100% of cells expressed the CD4 antigen (data not shown). Cells were PHA-stimulated for three days, infected with SIVmac251, and, again, viral replication was quantitated in supernatants by ELISA at five days post-infection. Again, results confirmed the potent inhibitory effect of raltegravir (Fig 1D).

We concluded that raltegravir inhibits SIVmac251 in different tissue culture assays at least with similar potency as observed in human primary cell-based assays [30,31]. The EC_95 _values are within the mean trough concentration (142 nM) measured in pharmacokinetic studies in humans [32].

### Raltegravir decreases viral load in SIVmac251-rhesus macaques and stably maintains suppressed viral loads when associated with RT inhibitors PMPA and FTC

**Figure 2:**
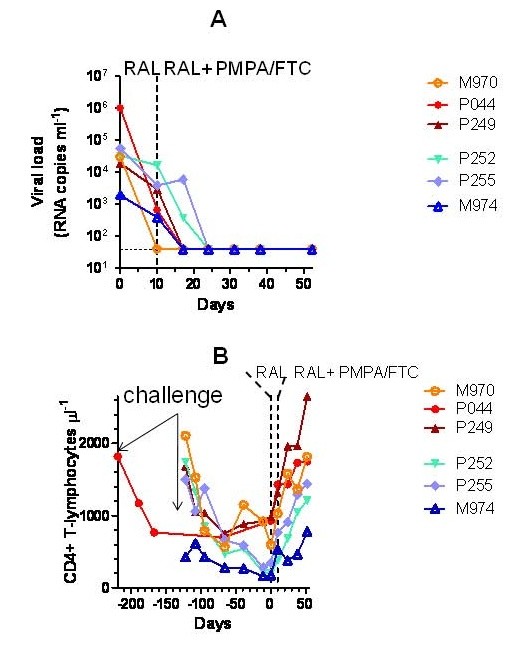
**Effect of raltegravir (RAL), alone and in combination with PMPA and FTC, on viral load (panel A) and CD4 counts (panel B) in SIVmac251-infected macaques (Group 1)**. SIVmac251-infected rhesus macaques (*Macaca mulatta*) were randomized to receive 50 (marked by the blue symbols) or 100 (red symbols) mg of raltegravir twice daily with food (bid). Monotherapy was continued for ten days. At day 11, nonhuman primates treated with 50 mg of raltegravir bid were switched to the 100 mg regimen, and two RT inhibitors, *i.e*. the NtRTI, tenofovir (PMPA) and the NRTI emtricitabine (FTC), were added to treatment (henceforth referred to as ART) in all animals. Viral load values positioning on the dotted line parallel to the *x *axis should read as undetectable.

To confirm susceptibility of SIVmac251 to raltegravir *in vivo*, we tested the effects of the drug in six rhesus macaques with stabilized infection by SIVmac251 (henceforth referred to as Group 1). The macaques had been challenged with SIVmac251 by either the rectal or vaginal route and were between 5 months and two years post infections prior to the start of raltegravir treatment. The macaques were randomized to receive 50 or 100 mg of raltegravir twice daily with food (bid). Monotherapy was continued for ten days. At day ten, raltegravir treatment resulted in a significant decrease in viral load (*P *= 0.031, Wilcoxon signed rank test) (Fig. 2A). The 100 mg treatment subgroup apparently had higher decreases in viral load than the 50 mg treatment subgroup, although the numbers of animals did not allow statistical evaluation of differences between subgroups. Of note, one animal treated with the 100 mg bid dosage showed an undetectable viral load (detection threshold: 40 copies of viral RNA ml^-1^). Virological response to raltegravir was associated with a significant increase in CD4 counts (*P *= 0.017, Wilcoxon signed rank test), detectable in all animals (Fig. 2B). We concluded that raltegravir-treated animals showed viro-immunological improvement.

This group of nonhuman primates had been released by another study showing that viral loads had been stable before initiating raltegravir treatment (data not shown). In the prior study, unfortunately, viral load had been measured by another technique (NASBA), thus rendering incorrect a possible statistical comparison between the historical values and the pre-and post raltegravir treatment values from the present study.

Comparison of the CD4 values after raltegravir monotherapy with historical data derived from flow-cytometric determination of CD4 numbers was instead possible. The data available from the time of SIVmac251 inoculations showed that the CD4 counts prior to raltegravir treatment had been gradually decreasing, or maintained at levels lower than pre-inoculation values, as a sign of the ongoing lentiviral infection [33]. Our results showed that raltegravir abruptly changed the trends in the CD4 counts (Fig. 2B). For five of the Group 1 animals, it was possible to make a multiple comparison between values at ten days prior to treatment start, at Day 0, and Day 10 of raltegravir monotherapy. Repeated-measures ANOVA reported an extremely significant difference (*P *= 0.0014). The CD4 counts post-monotherapy significantly deviated from values at Day 0 and ten days prior to raltegravir administration (*P *< 0.05 in both cases; Bonferroni's post-test for multiple comparisons), whereas no significant difference was found between values prior to treatment start and Day 0 (*P *> 0.05). We concluded that there was a significant association between CD4 rise and raltegravir treatment.

At day 11, nonhuman primates treated with 50 mg of raltegravir bid were switched to the 100 mg regimen (in order to prevent selection of drug-resistant mutants), and two RT inhibitors, *i.e*. the NtRTI, PMPA and the NRTI FTC, were added to treatment (henceforth referred to as ART) in all subjects. Results showed that viral load continued to decrease: an undetectable viral load was shown by four animals after one week, and by all study animals after two weeks (Fig. 2A). Viral load was maintained undetectable until the end of follow-up (Day 52). In parallel, CD4 counts continued to increase up to restoration of values at the time of inoculation (Fig. 2B). We concluded that the ART regimen based on raltegravir plus PMPA and FTC suppressed viral replication to undetectable levels in nonhuman primates and restored CD4 counts.

As expected from results in human clinical trials, therapy was well-tolerated from a clinical point of view, and serum chemistry (kidney and liver enzymes) and hematology values remained within normal limits (data not shown).

### The virological improvement of SIVmac251-infected animals is significantly associated with raltegravir treatment

The results in Group 1 nonhuman primates clearly show that raltegravir, and ART, induced viro-immunological improvement of nonhuman primates with progressing SIVmac251 infection.

**Figure 3:**
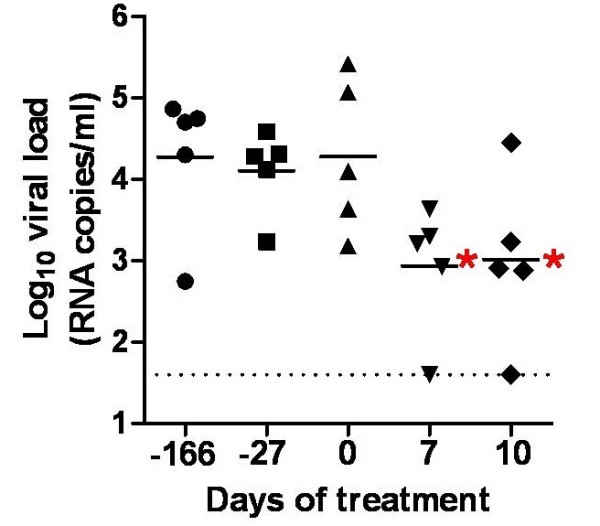
**Association of viral load decrease with raltegravir treatment of SIVmac251-infected animals (Group 2)**. SIVmac251-infected rhesus macaques (*Macaca mulatta*) received 100 mg of raltegravir twice daily with food (bid). Monotherapy was continued for ten days. Comparison between pre- and post-raltegravir viral load measurements was done. Viral load values at Day 0, Day 7 and Day 10 were compared with viral loads at 27 and 166 days prior to treatment start. Significant differences (*P *< 0.05; Bonferroni's test following repeated-measures ANOVA; shown in the graph by the red asterisks) were found between both the values at 166 and 27 days prior to treatment start and the values at Day 7 and Day 10 of treatment. No significant differences, instead, were found between the values at 166 days, or 27 days, prior to treatment, and the values at Day 0. The dashed line parallel to the *x *axis marks the detection threshold of the technique adopted.

To exclude that the viral load decrease observed during raltegravir treatment of Group 1 could be attributed to random fluctuations of SIVmac251 replication, or by spontaneous acquisition, by the non human primates, of the capacity to control viral replication, we treated another group of non-human primates for which historical data were available using the same technique for viral load measurement (Group 2). In this group, we also measured viral load at seven days of treatment, in order to minimize the effect of time-dependent, spontaneous viral fluctuations on the decrease in viral load. Fig. 3 clearly shows that no significant changes in viral load were observable in 166 days in the absence of drug treatment (*P *> 0.05, Bonferroni's post-test following repeated-measures ANOVA). Viral load, however, did significantly decrease in only seven days of raltegravir treatment (*P *< 0.05). Despite the small number of non-human primates enrolled, the *P *values obtained support the extreme significance of the anti-SIVmac251 effects of raltegravir. We concluded that 1) there was significant association between decreased viral load and raltegravir treatment, and that 2) the effects of raltegravir proved reproducible in two distinct groups of animals.

Again, one non-human primate in Group 2 showed an undetectable viral load following raltegravir monotherapy. This animal was the only component of Group 2 to show a low viral load (*i.e*., 1,520 copies/ml) before treatment was initiated. To further support the contribution of raltegravir treatment to the viral load decline in this subject, treatment was stopped and viral load was followed up. Results showed that a rebound in viral load occurred following treatment suspension (4,520 viral RNA copies/ml; value at two weeks from suspension).

### SIVmac251 proviral DNA persists during ART in peripheral blood mononuclear cells of the non-human primates

**Figure 4:**
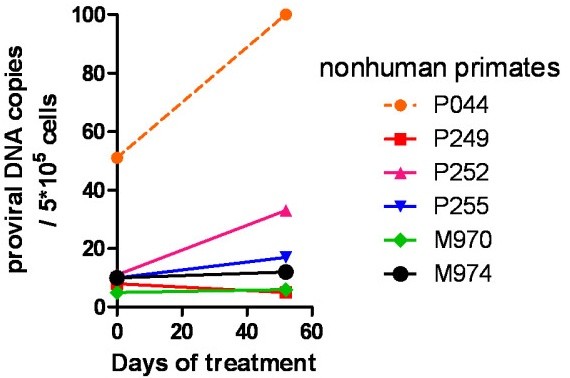
**Persistence of proviral DNA during therapy (Group 1)**. Proviral DNA was measured by a quantitative PCR technique at start of treatment with antiretroviral drugs, and at 52 days of therapy.

To evaluate whether copies of SIVmac251 proviral DNA persisted during ART despite suppression of viral load to undetectable levels, we measured proviral DNA copy numbers in PBMCs of the non-human primates prior to starting dosing and after 52 days of therapy. Results showed that proviral DNA was maintained stable during the treatment period analyzed. The difference between the proviral DNA levels at the two time points analyzed was not statistically significant (*P *> 0.05; Wilcoxon signed rank test) (Fig. 4). We concluded that ART regimens consisting of two NRTIs/NtRTIs plus raltegravir maintains stably suppressed SIVmac251 viral load, but not the proviral DNA, in non-human primates.

## Discussion

### Susceptibility of SIVmac251 to raltegravir

The results of the present study show that raltegravir inhibits SIVmac251 replication both in tissue culture and *in vivo*. The result is comparable to those of previous susceptibility studies using wild-type HIV-1 and HIV-2 [25,30] and is supported by similar assays conducted in the present study using HIV-1 and HIV-2 as positive controls for viral replication inhibition. The EC_50 _of raltegravir found by Hombrouck *et al*. [25] in the MTT-based assays for HIV-1 IIIB cythopathic effects is slightly lower than that obtained in the present study. Differences between our results and those of Hombrouck *et al*. can be attributed to the differences in the experimental protocols such as the higher MOI of HIV-1 used in the present study. Similarly, the higher EC_50 _of raltegravir for HIV-2 reported in a previous study of Roquebert *et al*. using HIV-2 ROD can be explained by the fact that these authors adopted a different method for viral quantification, *i.e*. a quantitative RT PCR assay [30]. On the other hand, the range of EC_95 _values obtained in the present study for HIV-1 overlap the 33 nM value reported previously, which became an acceptable threshold for the trough concentrations of the drug in pharmacokinetic studies [34].

The lower EC_50 _of raltegravir for the SIVmac251 cytopathic effect, as compared to that found in HIV-1-based assays, is likely to be attributed to the viral cytopathogenicity kinetics of SIVmac251 which is slower than that of HIV-1. Under our assay conditions, SIVmac251 required approximately fifteen days to kill the control untreated cultures, whereas HIV-1 only took five days. It is possible to hypothesize that the inhibitory effects of raltegravir in the SIVmac251-infected MT-4 cells subjected to prolonged treatment exposure is the result of the sum of the inhibition levels occurring during each of the multiple rounds of viral replication. When the EC_50 _was calculated on a viral antigen basis, the resulting values for SIVmac251 and HIV-1 were closer, because both sets of measurements were done at five days post-infection. This result is also confirmed by viral antigen capture assays using supernatants from primary PBMC and enriched CD4^+ ^cell fractions incubated under similar assay conditions.

Inhibition of SIVmac251 replication in tissue culture is in line with the declines in viral load obtained by raltegravir monotherapy of SIVmac251-infected non-human primates. Of course, factors other than drug treatment may have contributed to the viral load decline observed during treatment *in vivo*. For example, it has been shown that cytotoxic responses contributed to the viral load decline induced by another INSTI, the naphthyridine carboxamide, L-870,812 [21]. However, these responses in the absence of raltegravir could hardly control infection, as shown by the analysis of the CD4 counts of one of our study groups prior to treatment start. In this regard, the graph in Fig. 2B clearly shows that the nadir of CD4 counts was approximately coincident with Day 0 of raltegravir monotherapy. Subject M974 (belonging to this group) showed a low viral load (1,960 RNA copies/ml) at the beginning of treatment. However, this subject could not be regarded as an *élite *controller of the infection, because, prior to raltegravir administration, it also showed low CD4 counts (173 CD4^+ ^T cells/μl) which increased to 531 units/μl after 10 days of raltegravir monotherapy, and to 778 units/μl at 52 days of treatment with ART (Fig. 2A). Finally, the results obtained in another group of five macaques, for which historical viral load values were available prior to start of raltegravir treatment, showed that marked declines in viral loads were stringently associated to the period of raltegravir monotherapy. These results support the fundamental contribution of raltegravir administration to the antiretroviral effects. Moreover, after therapy suspension, a rebound in viral load was evident in an animal that had shown undetectable levels following raltegravir monotherapy. On the whole, these results show rapid virological and immunological response associated with administration of raltegravir in the simian AIDS model.

Although response to a naphthyridine carboxamide such as L-870,812 has already been assessed in the simian AIDS model, the susceptibility to raltegravir of SIVmac251 is far from obvious. Though mechanistically identical to L-870,812, raltegravir belongs to an unrelated chemical class, *i.e*. the *N*-alkyl-5-hydroxypyrimidinone carboxamides [35]. It has been well established that there may be discordant resistance between mechanistically identical INSTI drugs designed for HIV-1, and that non-human lentiviral enzymes often show structural differences to their HIV-1 counterparts mimicking specific drug resistance mutations [36,37]. In this context, the *in vivo *susceptibility of SIVmac251 to a further INSTI drug such as raltegravir supports the concept that the simian AIDS model responds to more than one class of INSTIs designed for HIV-1 and encourages pre-clinical testing of novel INSTIs in SIVmac251-infected nonhuman primates.

### Structural bases for the raltegravir response

An explanation for SIVmac251 susceptibility to raltegravir may be derived from comparison of the SIVmac251 IN with INSTI-susceptible or resistant HIV-1 INs; and, conversely, the data provided herein, using SIVmac251, may furnish novel insights into the understanding of the raltegravir response of HIV-1. Primary resistance to raltegravir has been associated with three major mutations, N155H, Q148H/K/R, and Y143H; mutation of any of these HIV-1 IN amino acids initiates pathways leading to raltegravir resistance [22,38,39]. These residues are located around the active site of IN and within interacting distance to raltegravir, as shown by molecular modelling simulations conducted by independent groups [27,40]. Drug resistance mutations N155H and Q148R were shown to hamper INSTI binding to HIV-1 IN, by either decreasing the affinity of IN/proviral DNA complexes for INSTIs (N155H) or affecting assembly of proviral DNA (Q148R) [41]. Secondary mutations reported for raltegravir are L74M, E92Q, T97A, E138K, G140S/A, V151I, G163R, I203M, S230R, and D232N [22,38,40].

**Figure 5:**
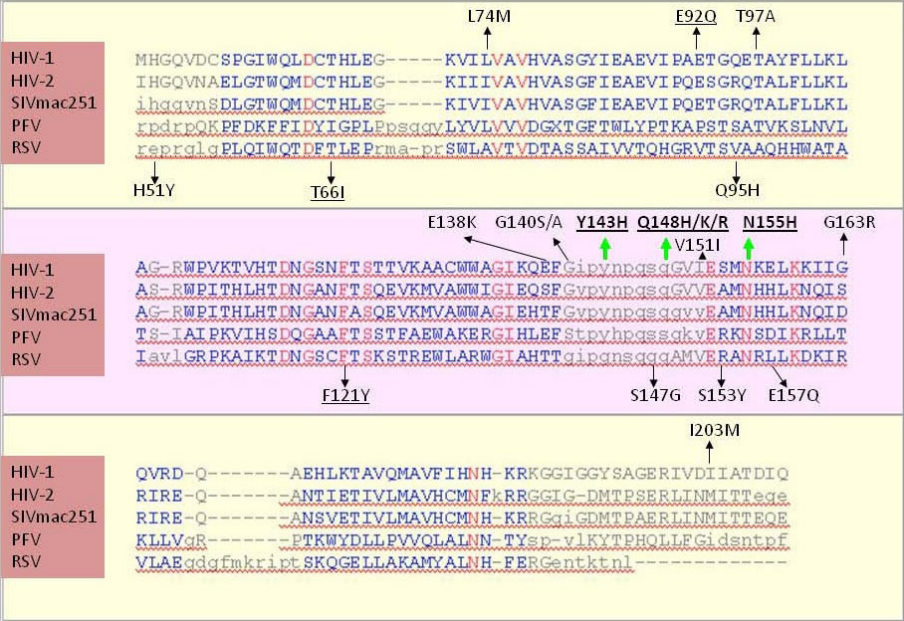
**Sequence alignment of the integrase catalytic core domains of HIV-1 subtype B (PDB: **1BL3_C**), HIV-2 (PDB: **3F9K_A**), SIVmac251 (PDB: **1C6V_A**), prototype foamy virus/PFV (PDB: **3DLR_A**), and Rous Sarcoma virus/RSV (PDB: **1ASU_A**)**. The sequence alignment is based on a structural alignment performed using the VAST algorithm. Regions showing significant structural alignment are presented in blue, with the highly conserved residues shown in red. Above the alignments are shown the mutations found in HIV-1 infected individuals failing raltegravir-based drug regimens (the green arrows indicate the primary resistance mutations Y143H, Q148H/K/R, and N155H; black arrows indicate secondary resistance mutations). Other drug resistance mutations induced by other integrase strand transfer inhibitors are shown below the alignments. The mutations shown by site-directed mutagenesis to confer resistance to raltegravir are underlined. Note that the structure for HIV-1 subtype B integrase catalytic core domain (**PDB: **1BL3_C) presents the secondary drug resistance mutation V151I.

According to structural alignments of the HIV-1 IN CCD with published structures of the IN CCDs from SIVmac251 and other retroviruses with reported profiles of susceptibility to INSTIs, we found that the amino acid positions corresponding to Y143, Q148, and N155 are conserved between HIV-1 and SIVmac251 (Fig. 5). These amino acids are also conserved in HIV-2 IN (susceptible to raltegravir [30]) but are not in prototype foamy virus (PFV; susceptible to raltegravir but showing EC_50 _values 1-2 orders of magnitude higher than the EC_50 _for HIV-1 [42]) or Rous sarcoma virus (RSV) IN (which is not inhibited by INSTIs designed for HIV-1 [26]). Several amino acids are also conserved between SIVmac251 and HIV-1 at positions susceptible to secondary drug resistance mutations. Among these, conservation of E92 is particularly relevant because, differently from other secondary resistance mutations, the E92Q mutation alone is capable to decrease raltegravir susceptibility in the absence of primary resistance mutations [43]. Instead, the amino acid corresponding to HIV-1 IN E92, is a proline in PFV and a valine in RSV.

Similar to HIV-2, SIVmac251 mimics polymorphisms at some of the secondary drug resistance positions in HIV-1 (L74, E138, G163 and I203). Among these, the only drug resistance mutation mimicked by SIV is I203M (Fig. 5). This mimicry, however, is shown also by HIV-2 IN, which, as mentioned above, is fully susceptible to raltegravir. Changes in this position may thus be irrelevant in the absence of primary drug resistance mutation Y143R/C [44]. Outside the IN CCD at the site corresponding to HIV-1 IN S230 (not shown in the sequence alignment of Fig. 5), SIVmac251 presents a glycine, which, however, does not mimic the corresponding drug resistance mutation S230R in HIV-1 IN.

Two drug resistance mutations induced by other INSTIs were shown to confer cross-resistance to raltegravir [43]. T66I is a primary drug resistance mutation raised by the investigational quinolone INSTI, elvitegravir, and some diketo acids [35,45]. F121Y is a primary drug resistance mutation for naphthyridine carboxamide L-870,810 [26]. The amino acids presented by SIVmac251 in these positions strictly correspond to those found in wild-type HIV-1 and HIV-2 INs (Fig. 5).

**Figure 6:**
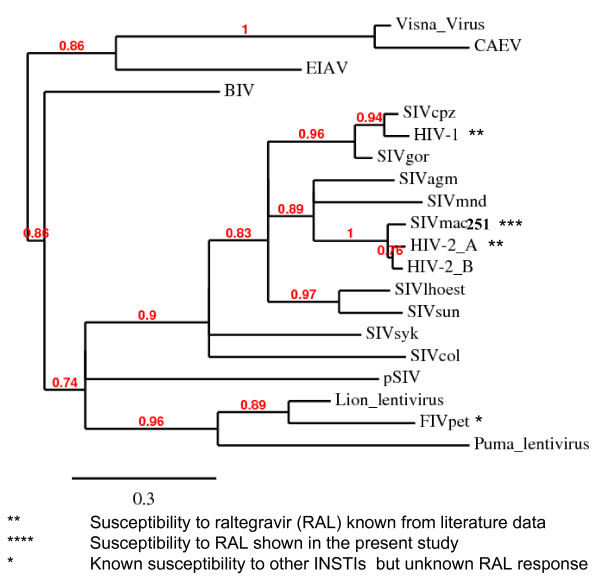
**Phylogenetic tree of lentiviral integrase core domains**. Sequences adopted: human immunodeficiency virus type-1 (HIV-1) [PDB: 1BL3C]; human immunodeficiency virus type-2 (HIV-2) [PDB: 3F9K*]*; simian immunodeficiency virus, host: macaque (SIVmac251) [PDB: 1C6VC]; simian immunodeficiency virus, host: chimpanzee (*Pan troglodytes*) (SIVcpz) [accession: AAF18575]; simian immunodeficiency virus, host: gorilla (*Gorilla gorilla*) (SIVgor) [accession: ACM63211]; simian immunodeficiency virus, host: African green nonhuman primate (*Chlorocebus sp*.) (SIVagm) [accession: CAA30658]; simian immunodeficiency virus, host: mandrill (*Mandrillus sphinx*) (SIVmnd) [accession: AAB49569]; simian immunodeficiency virus, host: *Cercopithecus lhoesti *(SIVlhoest) [accession: AAF07333]; simian immunodeficiency virus, host: Skyes' nonhuman primate (*Cercopithecus albogularis*) (SIVsyk) [accession: AAS97874]; simian immunodeficiency virus, host: Colobus nonhuman primate (*Colobus guereza*) (SIVcol) [accession: AAK01033]; prosimian immunodeficiency virus, host: *Microcebus murinus *(pSIV) [see: additional material in Ref. [46]]; feline immunodeficiency virus, host: domestic cat (*Felis sylvestris*) (FIV-Pet) [accession: AAB59937]; lion lentivirus, host: lion (*Panthera leo*) [accession: ABX25835]; puma lentivirus, host: mountain lion (*Puma concolor*) [accession: AAA67168]; caprine arthritis-encephalitis virus (CAEV), host: *Capra hircus *[accession: NP_040939]; visna lentivirus, host: sheep (*Ovis aries*) [PDB: 3HPG_A]; equine infectious anemia virus (EIAV) host: horse (*Equus caballus*) [accession: NP_056902]; bovine immunodeficiency virus (BIV) host: wild banteng (*Bos javanicus) *[accession: Q82851]. Relationships between proteins were reconstructed using Phylogeny.fr. Approximate likelihood ratios > 70% are shown. This tree is not intended to reconstruct the phylogeny of primate lentiviruses, but rather to highlight the degree of similarity of the IN CCDs derived from different viruses. The similarities shown are in line with previous phylogenetic analyses based on DNA sequences corresponding to other portions of the lentiviral genome [74].

If the known susceptibilities of different lentiviruses to raltegravir, or other INSTIs, are mapped to a phylogenetic tree of primate lentivirus IN CCDs (Fig. 6), SIVmac251 IN clusters with a clade comprising HIV-2 IN, which is distinct from, but adjacent to the cluster of primate lentivirus INs comprising HIV-1 IN (Fig. 6). A relatively recent common ancestor of HIV-1 and SIVmac251/HIV-2 INs may explain their common susceptibility to raltegravir. Of note, conservation of the key amino acids T66, E92, F121, Y143, G148 and N155 (determining susceptibility to raltegravir) is shared by all primate lentiviruses analysed and is displayed also by highly divergent primate lentiviruses, including SIVcol, SIVsyk and the endogenous lentivirus pSIV, recently identified by Gifford *et al*. in basal primate *Microcebus murinus *[see Additional file 3] and sharing intermediate characteristics between primate and feline lentiviruses [46].

**Figure 7:**
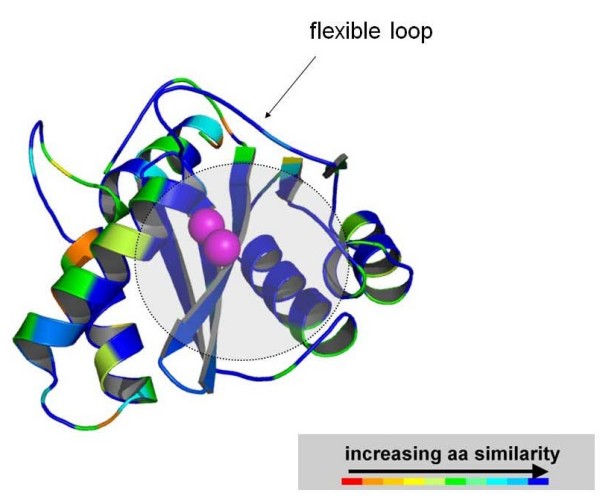
**A three-dimensional model of SIVmac251 IN catalytic core domain colored by amino acid similarity with wild-type HIV-1 IN**. The enzyme is coloured by sequence similarity with its HIV-1 orthologue [PDB:1BL3]. The level of similarity was calculated by the Swiss PDB Viewer (SPDBV) software. The colour scale is that adopted by SPDBV. Similarity is maximal at the level of the INSTI/cellular DNA binding site (indicated by a semi-transparent grey circle), as calculated by some of us in previous works [35]. Image obtained using PyMOL [73].

If the level of amino acid similarity between SIVmac251 and HIV-1 IN CCDs (calculated by the Swiss PDB Viewer program) is mapped to a 3D structure of HIV-1 IN CCD, it may be noted that amino acid identities cluster to the active site of IN, which is involved in INSTI binding [27,35] (Fig. 7). INSTIs bind at the interface between the IN active site and proviral DNA [1,2,47]. Modelling this interaction, however, has encountered several obstacles in the absence of crystallographic data for HIV-1 IN complexed with INSTIs, although several theoretical models for INSTI binding have been published so far [27,35,48-51]. A novel study using the "induced fit" docking (IFD) approach allowed conformational changes in the protein and DNA as well in order to obtain the best accommodation of the ligand [27]. Considering these findings, we built a SIVmac251 IN-Mg2^+^-DNA ternary complex as a target for IFD simulations of raltegravir binding [see Additional file 4].

**Figure 8:**
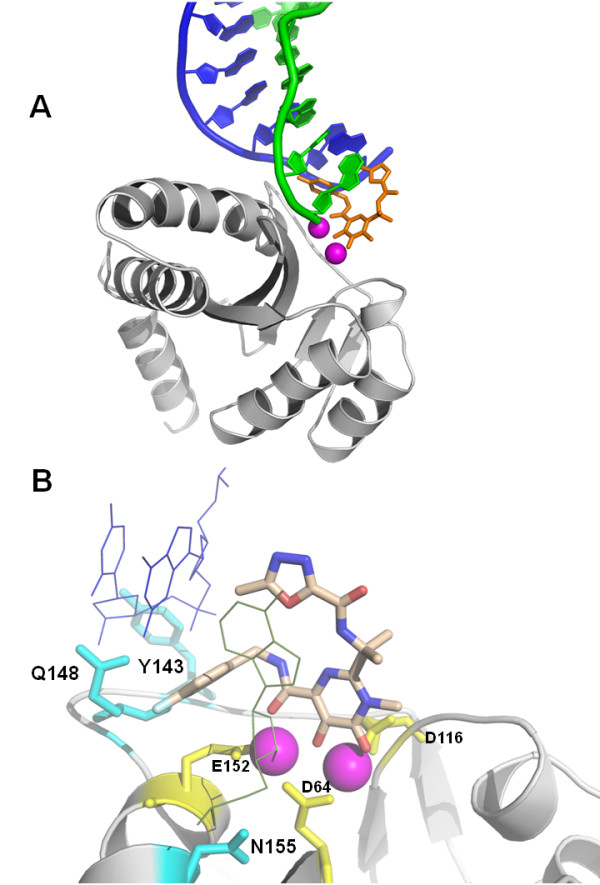
***In silico *docking of raltegravir at the SIVmac251 integrase (IN) active site**. Panel A: An overview of the interaction between SIVmac251 integrase (in grey), 3' processed proviral DNA (green and blue cartoons) and raltegravir (in orange). The three terminal nucleotides of the 5' DNA strand (in blue) have been removed for better clarity. Metal (Mg^2+^) ions are shown in magenta. Panel B: Interaction of raltegravir (shown in CPK) and the integrase amino acids susceptible to primary drug resistance mutations (cyan sticks). The protein backbone is shown by cartoons. Metal ions are presented in magenta. The catalytic triad (D64, D116 and E152) is shown in yellow. Ligand-interacting nucleotides, dC25 and dA20, are shown as thin lines. A full three-dimensional view of the complex can be obtained using the 3D coordinates provided as additional material [see Additional file 4]. Image obtained using PyMOL [73].

Only one IFD pose of raltegravir at the catalytic site of SIVmac251 (Fig. 7) came out from the IFD protocol, and it was similar to one of the two conformations of the drug at the HIV-1 IN catalytic site, as described in the previous IFD study [27]. This IFD pose clearly showed raltegravir as an ideal prosecution of the 3' DNA strand of 3'processed viral DNA, consistently with the hypothesis [52] that this drug acts as a nucleotide mimic (Fig. 8A). The three pharmacophoric oxygens of the drug were engaged in bidentate chelation of the two Mg^2+ ^ions within the catalytic cavity (Fig. 8B), while the substituted benzyl group deeply occupied a pocket mainly defined by IN residues Q148, E152 and H156, and viral nucleotides dG18, dC19, dA20, dG24 and dC25, as previously described in docking simulations at the HIV-1 IN CCD [27] [see Additional file 5]. Notably, during our docking simulations, the 3'-terminal adenine nucleotide dA20 underwent a dramatic conformational movement in order to allow insertion of the p-fluorobenzyl between the two viral DNA strands and a π-π interaction between the oxadiazole group and the 3' terminal adenine (Fig. 8B). It was also observed a possible cation-π interaction involving one metal ion and the aromatic tail of raltegravir (Fig. 8B).

We then analysed the positions of some key amino acids determining raltegravir susceptibility in the theoretical drug/target complex. The raltegravir docked conformations at the SIVmac251 and HIV-1 IN showed the aforementioned Q148 residue, important for drug susceptibility, as lying in close proximity to the ligand, *i.e*. within 2.2 Å (Fig. 8B). In particular, this residue shows strong van der Waals (vdW) interactions with the inhibitor (data not shown). No close contacts were observed, however, between N155 and raltegravir in both docking poses at the SIVmac251 and HIV-1 IN even if this residue is proximal to the ligand (*i.e*., within 5.5 Å). A recent study by researchers at Merck showed that the N155H mutation confers resistance to raltegravir primarily by perturbing the arrangement of active site Mg^2+ ^ions, thereby interfering with the chelating function of the inhibitor, and not by affecting the affinity of the metal or by affecting direct contacts of the inhibitor with the enzyme [53]. No close contacts were shown also for the third important amino acid determining susceptibility to raltegravir, *i.e*. Y143. The Y143 residue, however, showed a close interaction with the 5' strand of proviral DNA, which in turn is engaged in a close interaction (within 2.6 Å) with the methyl substituent on the oxadiazole moiety and the benzyl tail of raltegravir. If this docking pose is correct, it is possible to hypothesise that Y143 determines raltegravir susceptibility of SIVmac251 by maintaining DNA in a position allowing optimal drug/DNA interactions. As for the amino acids uninvolved in primary drug resistance, but shown to decrease susceptibility to raltegravir when mutated, F121 of SIVmac251 lies within 3.4 Å from the ligand, whereas E92 showed a significant shift from its original position following the IFD simulation (RMSD = 2.64 Å).

We also estimated the Gibb's binding energy (ΔG) of raltegravir complexed with SIVmac251 and HIV-1 INs and found a low percent difference observed between the two models (*i.e*. - 5.3%), which was consistent with the similar binding mode observed (data not shown). These chemoinformatic simulations support our experimental result showing that SIVmac251 is fully susceptible to raltegravir in tissue culture assays.

### A three-drug regimen to model lentiviral persistence during INSTI-based ART

Susceptibility of SIVmac251 to raltegravir is the basis for a novel antiretroviral treatment for non-human primates entirely based on drugs affecting the pre-integration stages of replication, and consisting of only raltegravir, and the two RT inhibitors (NtRTIs/NRTIs) PMPA and FTC. In our experiments, it was difficult to ascertain the contributions of the two drug classes to the achievement of an undetectable viral load in non-human primates. Addition of the NRTIs/NtRTIs to treatment was not intended to show a further contribution of these drugs to viral load suppression, since the effects, on SIVmac251, of both PMPA and FTC are well documented in literature [17]. Rather, PMPA and FTC at an early time point were obligate additions to therapy, in order to prevent drug resistance which occurs very frequently during monotherapy of lentiviral infections.

Since three-drug regimens consisting of raltegravir, tenofovir (*i.e*. the active form of PMPA) and FTC have become a new recommendable option for first-line therapy of HIV-1 as an alternative to NNRTI- or PI-based regimens, nonhuman primates subjected to this type of treatment may represent a valid alternative/complementary simian model to recently published models employing an RT-SHIV treated with two NRTIs and an NNRTI or a combination of two different simian lentiviruses treated with a mixture of different drugs including two PIs [20,23,54]. Response to ART was shown in all study subjects that we recruited, although they had been previously infected by different routes. This observation is in line with a previous study of ten Haaft *et al*., who investigated the effect of route of infection on early plasma viral RNA load in SIV(mac)-infected macaques. These authors found no significant difference in plasma RNA loads among the different routes of infection [55]. If this finding is confirmed in larger numbers, future studies requiring the ART-treated simian AIDS model might allow recruitment of nonhuman primates with extant infections and derived from other studies, *e.g*. controls for vaccine experiments, viral titration studies. This will allow shortening the experimental times and sparing economic resources and animal lives.

The ART-treated AIDS simian model described in the present study could be employed for preclinical evaluation of the effects of possible strategies for eliminating viral reservoirs or the testing of therapeutic vaccines. For example, an easy and rapid preliminary assessment of the impact of a candidate eradication strategy could be conducted by measuring the proviral DNA content of PBMCs. More sophisticated methods applicable to this model in order to quantify the effect of a therapy on lentiviral reservoirs could adopt limiting dilution techniques to detect the circulating CD4^+ ^T cells harboring replication-competent SIVmac251 or *in-situ *PCR from biopsies derived from tissues known to be viral sanctuaries. Finally, the effects of the candidate eradication therapy or the therapeutic vaccine treatment cycle could be shown by analyzing viral load rebounds, if any, after suspension of ART.

## Conclusion

We report that raltegravir is capable of inhibiting SIVmac251 replication both in tissue culture and *in-vivo*. This finding 1) supports the use of the simian AIDS models for pre-clinical testing of novel INSTIs for HIV-1 and HIV-2, and 2) is a basis for a new and effective ART regimen for the simian AIDS model entirely based on drugs adopted for treatment of humans. Our ART-treated AIDS nonhuman primate model could be employed to find possible strategies for combating lentiviral latency and eliminating reservoirs in attempts to eradicate the virus from the body.

## Methods

### Cells

We used the human MT-4 cells (T-CD4^+ ^cell line derived from cord lymphocytes transformed with HTLV-I virus, in which the proviral DNA is heavily methylated and produces no detectable virus) [56,57]. The CD4^+ ^CEMx174 cell line was also used. Cells were grown in RPMI-1640 medium supplemented with glutamine (200 mg/ml) (Invitrogen Life Technologies, Inc. Carlsbad, California), 10% heat-inactivated foetal bovine serum (FBS; Invitrogen Life Technologies), penicillin (500 U/ml; Pharmacia Italia SPA) and streptomycin (66.6 U/ml; Bristol-Myers, Sermoneta, LT).

Rhesus PBMCs were Ficoll-separated, resuspended at a concentration of 10^6^/ml and stimulated for 3 days with 5 μg/ml phytohaemoagglutinin (Difco Laboratories, Detroit, MI, USA) and 50 units/ml of human recombinant IL-2 (Roche Diagnostics, Indianapolis, IN, USA).

Rhesus CD4^+ ^T-cells were purified using magnetic-bead-based commercial kits (Miltenyi Biotec, Bergisch Gladbach, Germany), and then incubated for three days prior to infection under similar conditions as those adopted for stimulation of rhesus PBMCs.

### Virological assays

SIVmac251, HIV-1 (IIIB) and HIV-2 (CDC 77618) stocks were from the viroteques of the Italian of Institute of Health (Rome). Cells were infected for 2 h with the viruses at a multiplicity of infection of, approximately, 0.1, according to a protocol widely validated in our hands [57,58]. Cells were then washed three times in phosphate buffered saline, and suspended at 5 × 10^5^/ml in fresh culture medium (to primary cells 50 units/ml of IL-2 were added) in 96-well plates (Nunc, Roskilde, Denmark), in the presence or absence of a range of triplicate raltegravir concentrations (0.0001-1 μM) (Sigma, St Louis, MO, USA). Untreated infected and mock-infected controls were prepared too, in order to allow comparison of the data derived from the different treatments. Viral cytopathogeniciy in MT-4 cells was quantitated by the methyl tetrazolium (MTT) method (MT-4/MTT assay) when extensive cell death in control virus-infected cell cultures was detectable microscopically as lack of capacity to re-cluster. The capability of MT-4 cells to form clusters after infection was assessed as previously described [57]. Briefly, clusters were disrupted by pipetting; and, after 2 h of incubation at 37°C, the formation of new clusters was assessed by light microscopy (100 × magnification). Though not strictly quantitative, this method is highly sensitive, and has been repeatedly used in order to detect reproducible antiviral activity of compounds. Cell culture supernatants were collected for HIV-1 p24 and HIV-2/SIVmac251 p27 core antigen measurement by ELISA (Innogenetics N.V., Gent, Belgium; Advanced Bioscience Laboratories, Inc., Kensington, MD). In CEMx174-infected cell cultures, which show a propensity to form syncytia induced by the virus envelope glycoproteins [58], syncytia were counted, in blinded fashion, by light microscopy for each well at 5 days following infection.

### Nonhuman primate studies

#### Animals and drug treatments

The Indian Rhesus macaques used in this study were housed at BIOQUAL, Inc. Rockville, MD, according to standards and guidelines as set forth in the Animal Welfare Act and *The Guide for the Care and Use of Laboratory Animals*, as well as according to animal care standards deemed acceptable by the Association for the Assessment and Accreditation of Laboratory Animal Care International (AAALAC). All experiments were performed following institutional animal care and use committee (IACUC) approval. The macaques were inoculated mucosally, either intrarectally or intravaginally, with 300 MID_50 _(50% macaque infectious dose) of highly pathogenic SIVmac251. All macaques were infected and reached peak viral loads by week 2 and set point by week 12. Raltegravir was dosed by the oral route, either 50 mg/kg/BID or 100 mg/kg/BID.

PMPA [(R)-9-(2-phosphonylmethoxypropyl) adenine] and FTC {5-fluoro-1-(2R,5S)- [2-(hydroxymethyl)-1,3-oxathiolan-5-yl]cytosine} were kindly provided by Gilead Sciences through a material transfer agreement. Animals were doses subcutaneously with PMPA, 20 mg/kg/day, and FTC, 50 mg/kg/day.

#### Quantitative assay for SIVmac251 viral RNA levels

For measurement of plasma SIVmac251 RNA levels, a quantitative TaqMan RNA reverse transcription-PCR (RT-PCR) assay (Applied Biosystems, Foster City, Calif.) was used, which targets a conserved region of *gag *and has an accurate detection limit. The sensitivity of the method is two copies per run, which results in a detection limit as low as 40 RNA copies/ml. The samples were then amplified according to a method previously validated in our hands [59,60]. Briefly, a 500-μl aliquot of plasma was spun down at 13,000 × *g *for 1 h. The liquid was poured off and 1 ml of RNA-STAT 60 was added. After 5 min., 250 μl of chloroform was added and vortexed. The samples were spun at the same speed for 60 min. The clear aqueous layer on top was removed, and added to 500 μl of isopropanol. Then, 10 μl of 10 μg/ml tRNA was added and precipitated overnight at -20°C. The samples were spun for one hour, washed with a cold (-20°C) 75% ethanol solution, and re-spun for 60 minutes. The RNA was resuspended in 30 μl of RNAse-free water. 10% of the resuspended RNA was added to Taqman reagents (Applied Biosystems), plus primers and probe, and amplified in a 7700 Sequence Detection System by Applied Biosystems. Briefly, the sample was reverse transcribed at 48 degrees for 30 min. using One-Step RT-PCR Master Mix (Applied Biosystems), then held at 95°C for 10 min., and run for 40 cycles at 95°C for 15 sec. and 60°C for 1 min. The following PCR primer/probes were used: SIV2-U 5' AGTATGGGCAGCAAATGAAT 3' (forward primer), SIV2-D 5' GGCACTATTGGAGCTAAGAC 3' (reverse primer), SIV-P 6FAM-AGATTTGGATTAGCAGAAAGCCTGTTGGA-TAMRA (TaqMan probe). The signal was finally compared to a standard curve of known concentrations from 10^7 ^down to 1 copy (the linear range of concentration/signal relation spans eight *Log*s). All samples were done in triplicate for consistency and accuracy.

#### Quantitative assay for SIVmac251 proviral DNA

For proviral DNA detection, cells were spun down to a pellet, and the supernatant was poured off. The cell pellet was lysed with 1 ml of DNASTAT for 10 min. 250 μl of chloroform was added and the mixture was vortexed. The samples were spun at 13,000 for 60 min. and the aqueous layer was removed and added to another tube. To this, 500 μl of isopropanol was added, and the mixture was precipitated overnight at -20°C. The samples were then spun for one hour and the precipitate was washed with a -20°C-cold, 75% ethanol solution, and re-spun for 60 min. The DNA pellet was resuspended in 30 μl of water and 10% of the resulting solution was added to Taqman reagents (Applied Biosystems) plus primers and probe (the same as in previous paragraph) and amplified in a 7700 Sequence Detection System by Applied Biosystems. The signal was finally compared to a standard curve of known concentrations from 10^6 ^down to 1 copy (the linear range of concentration/signal relation spans seven *Log*s). The detection limit of this assay is two copies of proviral DNA/5 × 10^5 ^cells.

#### Flow cytometry

Hematology was performed by IDEXX (IDEXX Preclinical Research, West Sacramento, CA). For calculation of absolute cell numbers, whole blood was stained with anti-CD3-fluorescein isothiocyanate (FITC)/anti-CD4-phycoerythrin (PE)/anti-CD8-peridinin chlorophyll α protein (PerCP)/anti-CD28-allophycocyanin (APC), and anti-CD2-FITC/anti-CD20-PE, and red blood cells were lysed using lysing reagent (Beckman Coulter, Inc., Fullerton, Calif.). Samples were run on a FACSCalibur (BD Biosciences, San Jose, CA).

### Statistical analyses

Data were analysed using the software GraphPad Prism 5.00.288 (GraphPad Software, Inc., San Diego, CA). For calculation of the EC_50_, EC_90_and EC_95 _values, data were transformed into percentage-of inhibition values, plotted on *x*, *y *graphs, and subjected to linear or non-linear regression, depending on the best-fitting equation.

The numbers of animals enrolled in each treatment group were determined using the free-access online calculator for the β-error embedded in the DSS Research website [61].

For calculation of *P *values for changes in viral load and immunological parameters, pre- and post-monotherapy values were analysed using the Wilcoxon signed rank test. For multiple comparisons at different time points, data were analyzed by repeated-measures ANOVA followed by Bonferron's post test for comparison between the different experimental time points. An appropriate transformation was done to restore normality, where necessary.

### Bioinformatic analyses

Structural alignments of the catalytic core domains (IN CCDs) of lentiviral integrases were retrieved by the VAST algorithm embedded in the US National Center for Biotechnology Information (NCBI) website. Cn3D 4.1 (downloadable from the NCBI website) was used to visualize the superimposed three dimensional (3D) structures and the structure-based sequence alignments.

The Swiss PDB Viewer (SPDBV) program (Swiss Institute of Bioinformatics) was used to colour the 3D structures by alignment diversity. Briefly, the α-carbons of the highly conserved catalytic triads (*e.g*. D64, D116 and E152 for HIV-1 IN) were initially superimposed using the "fit molecules" option. Then, using the "improve fit" option, SPDBV was asked to minimize the root-mean square distance (RMSD) between the corresponding atoms using a least square algorithm. Using the default matrix embedded in the program (with open and extended gap penalties of 6 and 4, respectively), the calculation was extended to neighbouring atoms until the maximum number of aligned atoms with the lowest RMSD was obtained. Then, the "colour-alignment diversity" option was used. The coloured structures were then reconstructed manually using Pymol (DeLano Scientific, Palo Alto, CA), which generates higher-quality images.

Phylogenetic trees were generated using the *Phylogeny.fr *website [62,63], which, following a predefined pathway using MUSCLE [64], Gblocks [65], PhyML [66] and TreeDyn [67] outputs the corresponding phylogenetic tree.

### Molecular modeling

Recently, a HIV-1 IN-Mg^2+^-DNA model that mimics the product of 3' processing has been reported [27,68]. This complex has now been used as template to build the analogous SIVmac251 two metal-IN-DNA complex, starting from the available coordinates of the CCD in the RCSB Protein Data Bank. Indeed, 1C6V [24] is the crystal structure of SIVmac251 IN that contains four core domains (chains A-D) and one C-terminal domain (chain X); all the CCDs show no metal ions in the catalytic site and have one unresolved region (residues 141-151) mostly corresponding to the flexible loop (residues 140-149) adjacent to the active site.

In order to build our model, we used the molecular modelling package Schrödinger Suite 2007 (Schrödinger, LLC, New York, NY).

The SIVmac251 IN-Mg^2+^-DNA ternary complex was thus developed by superimposing the backbone Cα atoms of the three catalytic residues in SIVmac251 and HIV-1 integrases (D64, D116, and E152); in particular, we used the chain A of SIVmac251 IN, while chains B-D and X were discarded.

The unresolved region in the SIVmac251 CCD was then computationally completed based on the conformation of the homologous region in HIV-1 CCD. The coordinates of the two Mg^2+ ^ions and of the viral DNA in the HIV-1 model were also spliced into the SIVmac251 CCD, leading to a two metal model of IN-DNA complex. The nucleotide dT14 was manually corrected to dC14 in line with the cytosine presented in this position by sooty mangabey-derived viruses (GenBank accession: L26023). Examination of the catalytic triad highlighted that D64 and D116 side chains did not have the right conformation for favourable interaction with the Mg^2+ ^ions at the active site; thereby, their geometries were modified to metal-coordinating position.

Before the docking run, the complex was submitted to Schrödinger's Protein Preparation Wizard: water molecules were deleted, hydrogen atoms were added, bond orders and charges were then assigned, the orientation of hydroxyl groups on Ser, Thr and Tyr, the side chains of Asn and Gln residues, and the protonation state of His residues were optimized.

To remove the worst contacts between the parts of this new structure but not to alter the architecture of the binding site, 100 steps of steepest descent minimization (OPLS-2005 force field) using GB/SA model [69] as solvation treatment were carried out by freezing the two cations and the oxygen atom of the 3'OH of adenosine-viral DNA.

The structure of raltegravir was constructed using the Schrödinger Maestro interface and was then submitted to Polak-Ribiere conjugate gradient minimization [0.0005 kJ/(Å mol) convergence]. The phenolic oxygen of the ligand was considered as phenolate given the influence of the two metal ions in the binding site.

The Induced Fit Docking (IFD) protocol [70] was then employed in this study to accurately predict ligand binding modes and concomitant structural changes in the receptor. Briefly, IFD methodology merges the docking and scoring capabilities of program Glide with a protein structure prediction and refinement module (Prime) to generate reasonable binding structures for ligands known to be active but unable to be docked in an existing structure of the receptor using the rigid approach.

The IFD protocol used in this study was carried out using the following steps (the description below is from the IFD manual):

1. Constrained minimization of the receptor (Glide protein preparation, refinement only) with an RMSD cutoff of 0.18 Å;

2. Initial Glide docking of each ligand using a softened potential (van der Waals radii scaling). By default, a maximum 20 poses per ligand are retained, and by default, poses to be retained must have a Coulomb-vdW score <100 and an H-bond score <-0.05;

3. One round of Prime side chain prediction for each protein/ligand complex, on residues within a given distance of any ligand pose (6 Å in our study);

4. Prime minimization of the same set of residues and the ligand for each protein-ligand complex pose. The receptor structure in each pose now reflects an induced fit to the ligand structure and conformation;

5. Glide redocking of each protein/ligand complex structure within a specified energy of the lowest-energy structure (default: 30 kcalmol^-1^). The ligand is now rigorously docked, using default Glide settings, into the induced-fit receptor structure;

6. Estimation of the binding energy (IFDScore) for each output pose.

In our study, all docking calculations were run in the "Standard Precision" mode of Glide, and the center of the grid box was defined by the manually selected Mg ions.

The IN/DNA/ligand complex obtained by the IFD protocol was minimized performing a Polak-Ribiere conjugate gradient unrestrained minimization [0.005 kJ/(Å mol) convergence], using the OPLS-2005 force field [71] and the GB/SA model as solvation treatment [69].

Prime was then used to estimate the free binding energy (ΔG) of HIV-1 and SIVmac251 INs bound to raltegravir, using the MM-GBSA method [72]; OPLS-2001 was used as all-atom molecular mechanics force field [71] and GB/SA as solvation treatment [69].

## Competing interests

The authors declare that they have no competing interests.

## Authors' contributions

MGL, assisted by MC, coordinated the *in vivo *experiments in nonhuman primates. SN, participated at the bioinformatic analyses, and generated the tissue culture data on SIVmac251 susceptibility to raltegravir. MLB and NI built the SIVmac251/IN/Mg^2+^/DNA model, conducted the molecular docking simulations and measured the theoretical binding energies of the complexes. BC participated in the generation of in tissue culture data on drug susceptibility. JYO and JG, respectively, organized and prepared the *ex vivo *plasma and PBMC sampling, and conducted the quantitative PCR assays. FT cultivated and titrated the virus for the tissue culture experiments. EG conceived the study together with AS and helped AS in the study coordination. AS conceived and coordinated the study, did the experimental design, supervised and participated at the generation of *in vitro *data, conducted the bioinformatic and statistical analyses and drafted the manuscript.

## Supplementary Material

Additional file 1 **Structural alignment of the integrase catalytic core domains (IN CCDs) HIV-1 subtype B (PDB: **1BL3**) and SIVmac251 (PDB: **1C6V**)**. The alignment was conducted on structures deposited in the NCBI database using the VAST algorithm embedded in the website. The structures were then visualised using Cn 3D v. 4.1 (available freely from NCBI). The video was created using SnagIt (TechSmith Corporation Okemos, MI). The HIV-1 and SIVmac251 CCDs are shown in violet and blue, respectively. The active site is shown by the highly conserved catalytic residues D64, D116 and E152 (presented in yellow) and by the Mg^2+ ^ion coordinated by D64 and D116 in the 1BL3 structure. The flexible loop (residues 140-151) is not present in the alignment, due to its variable conformation that may not correspond to that adopted in vivo when the IN CCD is complexed with proviral DNA. The corresponding sequence alignment is shown in Fig. 5. Click here for file

Additional file 2 **Correlation between inhibition of p24 production and inhibition of syncytium formation in acutely HIV-1-infected CEMx174 cells**. Cells were infected with HIV-1 (IIIB), washed and incubated for five days in the presence or absence of a range of concentrations of raltegravir in a 96-well plate. HIV-1 p24 was quantified in supernatants by commercially available ELISA kits. The numbers of syncytia per well were determined by light microscopy in blinded fashion. Data from one representative experiment are shown and presented as the percentage of inhibition occurring at each of the tested concentrations of raltegravir. The concentrations to which the different data points refer are indicated by arrows in the graph. The solid line is the line best fitting the data points, as calculated by the least-squares method. Dashed lines mark the 95% confidence limits of the regression line. Statistical analysis reported an extremely significant correlation between the percentage-of-inhibition values calculated by the two different methods (*r *= 0.98; *P *= 0.0003; *t*-test for correlation). Click here for file

Additional file 3 **Sequence alignment of the integrase catalytic core domains from several lentiviruses**. For the sequences adopted, see caption of Figure 6. Click here for file

Additional file 4 **Three-dimensional coordinates of a theoretical model for raltegravir docking at the SIVmac251 integrase/proviral DNA interface**. Three-dimensional coordinates of a theoretical model for raltegravir docking at the SIVmac251 integrase/proviral DNA interface Click here for file

Additional file 5 **IFD binding mode of raltegravir at the SIVmac251 catalytic site in complex with proviral DNA**. Molecular surfaces are shown for IN (gray), catalytic loop (residues 140-149; cyan), metal ions (magenta), 3'-DNA strand (green), and 5'-DNA strand (yellow). This figure was prepared using PyMOL [73]. Click here for file
