# Supplementary figures and images for: Response of a simian immunodeficiency virus (SIVmac251) to raltegravir: a basis for a new treatment for simian AIDS and an animal model for studying lentiviral persistence during antiretroviral therapy
Source: Retrovirology. 2010 Mar 16;7:21. doi: 10.1186/1742-4690-7-21 (PMC2853490; doi:10.1186/1742-4690-7-21)

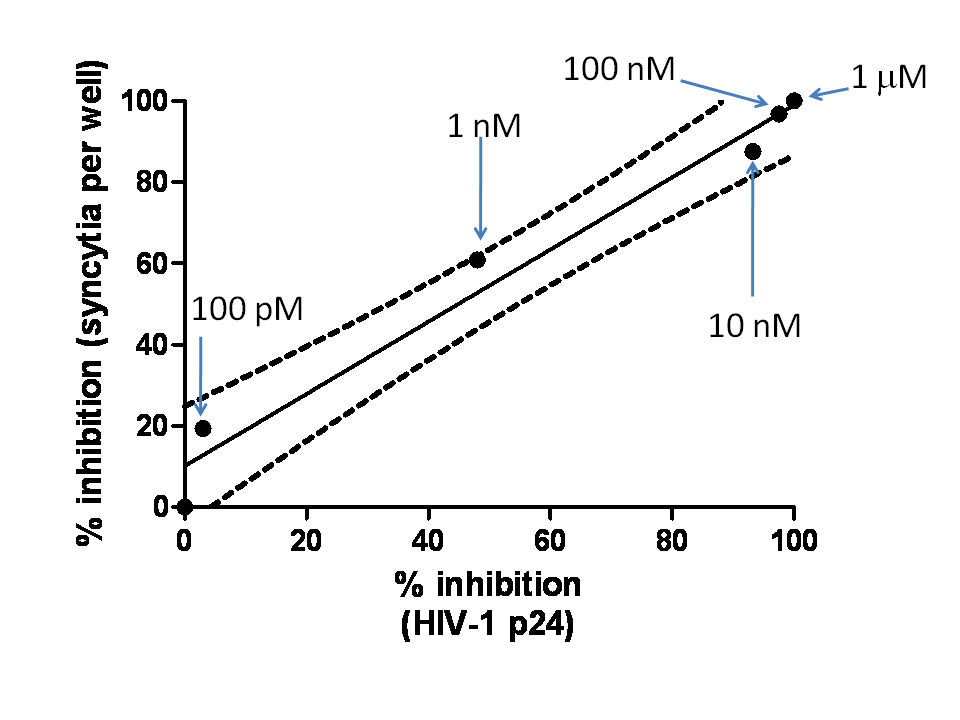

Supplement: Additional file 2 — Correlation between inhibition of p24 production and inhibition of syncytium formation in acutely HIV-1-infected CEMx174 cells. Cells were infected with HIV-1 (IIIB), washed and incubated for five days in the presence or absence of a range of concentrations of raltegravir in a 96-well plate. HIV-1 p24 was quantified in supernatants by commercially available ELISA kits. The numbers of syncytia per well were determined by light microscopy in blinded fashion. Data from one representative experiment are shown and presented as the percentage of inhibition occurring at each of the tested concentrations of raltegravir. The concentrations to which the different data points refer are indicated by arrows in the graph. The solid line is the line best fitting the data points, as calculated by the least-squares method. Dashed lines mark the 95% confidence limits of the regression line. Statistical analysis reported an extremely significant correlation between the percentage-of-inhibition values calculated by the two different methods (r = 0.98; P = 0.0003; t-test for correlation). [file 1742-4690-7-21-S2.TIFF]

## Slide 1
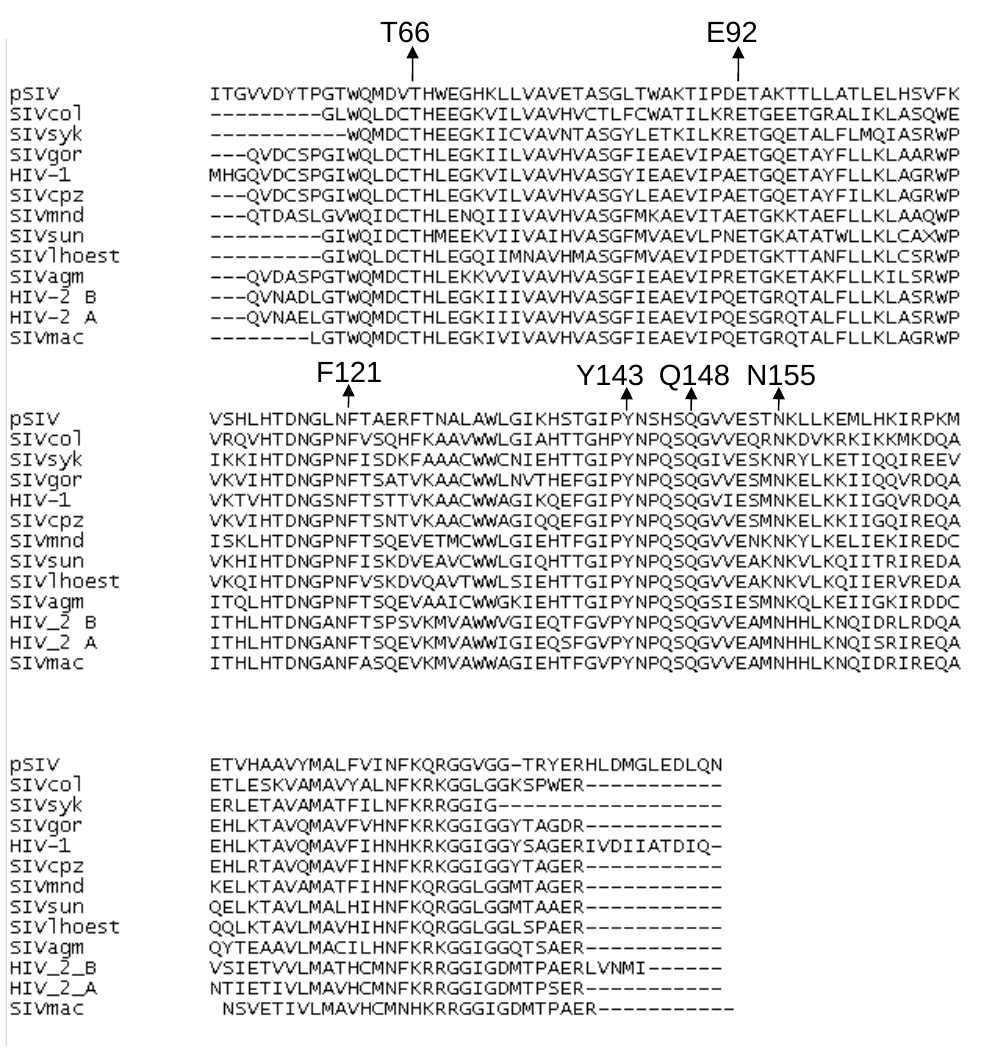

T66
E92
F121
Y143
Q148
N155

Supplement: Additional file 3 — Sequence alignment of the integrase catalytic core domains from several lentiviruses. For the sequences adopted, see caption of Figure 6. [file 1742-4690-7-21-S3.PPT]

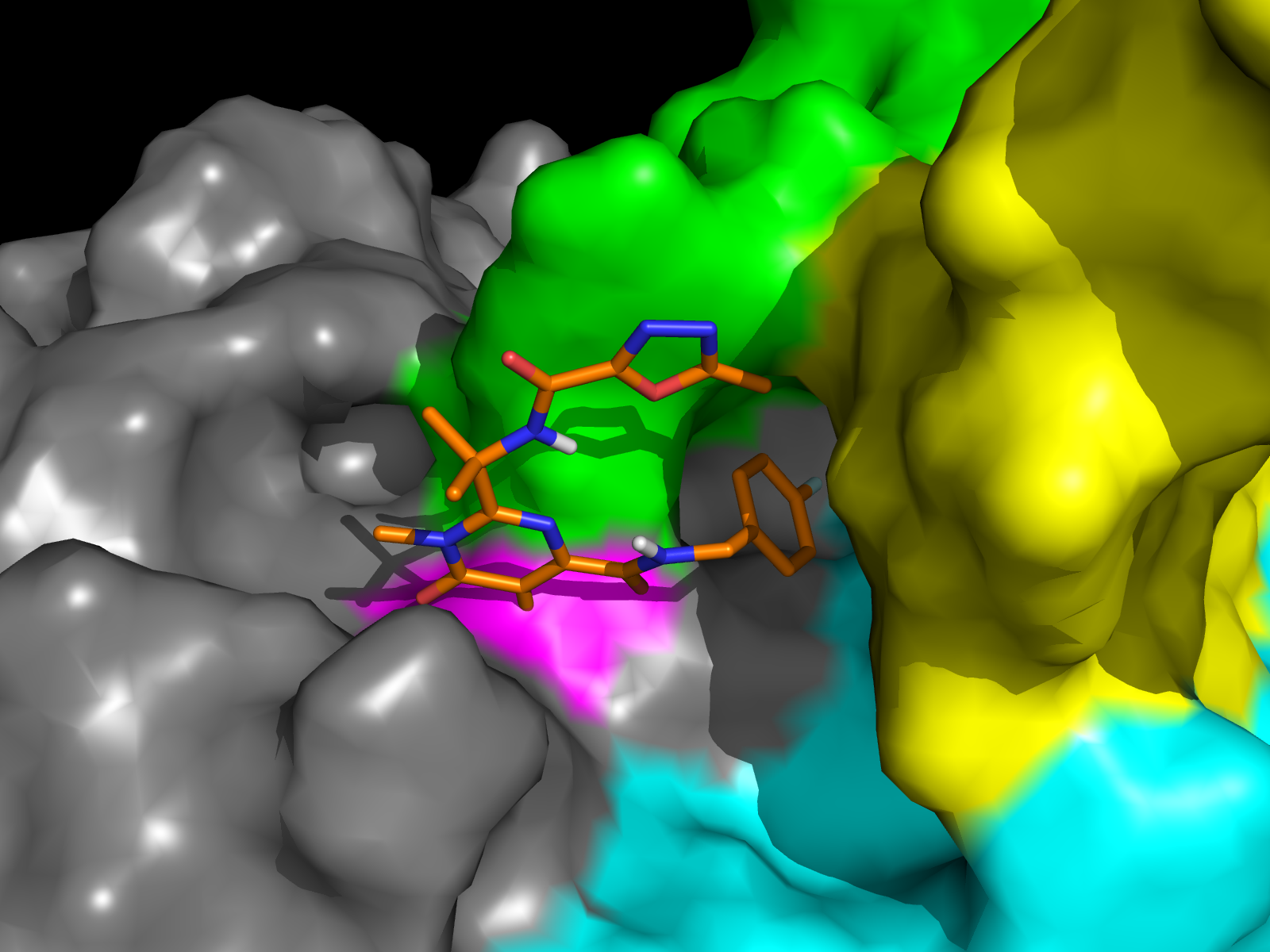

Supplement: Additional file 5 — IFD binding mode of raltegravir at the SIVmac251 catalytic site in complex with proviral DNA. Molecular surfaces are shown for IN (gray), catalytic loop (residues 140-149; cyan), metal ions (magenta), 3'-DNA strand (green), and 5'-DNA strand (yellow). This figure was prepared using PyMOL [73]. [file 1742-4690-7-21-S5.PNG]
